# KIF26B in the Prognosis and Immune Biomarking of Various Cancers: A Pan-Cancer Study

**DOI:** 10.1155/2022/4829697

**Published:** 2022-03-22

**Authors:** Fei Sun, Yingying Lian, Jianlin Wang, Lijun Hu, Judong Luo, Jingping Yu

**Affiliations:** ^1^Department of Radiotherapy, The Affiliated Changzhou Second People's Hospital of Nanjing Medical University, Changzhou 213003, Jiangsu Province, China; ^2^Department of Radiotherapy, Shuguang Hospital, Affiliated to Shanghai University of Chinese Traditional Medicine, Zhang Heng Road, Pudong New Area, Shanghai 201203, China

## Abstract

KIF26B has been identified as an oncogene in several tumors; however, its utility as a prognostic indicator for various cancers has not yet been comprehensively evaluated. Here, we first examined how KIF26B intervenes in thirty-three cancers within the TCGA database, including potential immunological functions, and how it affects the prognosis. Based on the open databases TCGA, TIMER2, GEPIA2, GTEx, CPTAC, and HPA, we found that, when compared with normal tissues, KIF26B is overexpressed in 22 tumor tissues. Following a survival analysis, a relationship between the expression of KIF26B and the prognosis of various cancers was observed. Among the genetic alterations assessed, mutations were the most frequent. On the contrary, high phosphorylation levels of S977 were detected in breast cancer, KIRC, LUAD, and UCEC. We also found positive or negative correlations between KIF26B and the immune infiltration of endothelial cells and cancer-associated fibroblast infiltration. This could imply that patients may benefit from immunotherapy. Finally, KEGG pathways and GO enrichment analyses were implemented to identify the molecular mechanisms of KIF26B. This study illustrates the function of KIF26B from a pan-cancer perspective and offers a new horizon for cancer prognostic and immunotherapeutic investigations.

## 1. Introduction

With the rapid development of genomics and proteomics, an increasing number of oncogenes and antioncogenes have been discovered. Tumors are generated by complex mechanisms, and a single gene may trigger various malignant growths. Thus, it is urgent to review the most relevant information on several cancer models to reach rigorous pan-cancer conclusions.

Member 26B of the kinesin family (KIF26B) has renowned functions on the adhesion and polarization of mesenchymal cells [1]. KIF26B has been identified as an oncogene in breast, gastric, colorectal, and hepatocellular cancers [2–5]. Moreover, the overexpression of KIF26B in these cancers has been relatively correlated with larger tumor size, higher risk of metastases, and poor prognosis.

Increasing evidence indicates that carcinogenesis and cancer progression are closely related to the tumor immune microenvironment (TME) [6]. Thus, prognosis and survival biomarkers associated with TME seem crucial to direct immunotherapy alone or combined with basic antitumor therapy [8]. Therapies targeting TME in combination with chemotherapy or radiotherapy have been applied in clinical treatments; however, their effectiveness and the mechanisms involved have not been fully studied [7].

Comprehensive bioinformatics analyses revealed that the members of the kinesin superfamily (KIFs) could potentially act as biomarkers and therapeutic targets in breast [9] and colorectal cancers [4]. KIF26B was also regarded as the posterior target of the Wnt5a-Ror pathway, which acts to regulate cytoskeleton-associated developments, for instance, cell migration, polarization, and adhesion [10, 11].

Despite the above, little is as yet known about the connection between KIF26B and cancer, and the pertinent studies are limited to a particular type, as well as a specific mechanism. In this context, a pan-cancer investigation of KIF26B through various cancers seems necessary. For this purpose, we implemented multiple bioinformatics approaches that evaluated gene expression, survival, genetic alteration, protein phosphorylation, endothelial cell immune infiltration, fibroblast infiltration, and pathway enrichment. Our results showed the prognostic and immunological value of KIF26B.

## 2. Materials and Methods

### 2.1. Expression of the KIF26B Gene

The mRNA levels of KIF26B in a variety of cancers were analyzed through the TIMER2 website (version 2 of the Tumor Immune Estimation Resource; http://timer.cistrome.org/). The GEPIA2 web server (version 2 of the Gene Expression Profiling Interactive Analysis; http://gepia2.cancer-pku.cn/#analysis) was employed to analyze the KIF26B mRNA levels in tumors with no corresponding normal tissues (TCGA-DLBC, TCGA-SKCM, etc.). The range was set as follows: log2 FC (log2 fold change) ≥ 1 or ≤ −1 and *P* value ≤ 0.01. To this end, comparisons were made with nontumor tissues from the GTEx (Genotype-Tissue Expression) records. Additionally, the relationship between KIF26B and pathological stages of cancers was observed using the “Stage Plot” of GEPIA2.

The protein expression profiles of human KIF26B in healthy and tumor tissues were compared through the open database HPA (http://www.proteinatlas.org/) [12]. Moreover, immunohistochemistry (IHC) staining images of the KIF26B protein were downloaded from HPA.

UALCAN is an interactive, user-friendly, and synthetic online platform that allowed the analysis of open-source TCGA cancer data [13]. Protein expression analyses were profiled with the Confirmatory/Discovery tool of the Clinical Proteomic Tumor Analysis Consortium (CPTAC) [14]. For its part, the phosphoprotein levels of KIF26B were assessed by CPTAC analysis.

### 2.2. Prognosis and Survival

The “Survival Map” component of GEPIA2 was applied to detect the OS (overall survival) and the DFS (disease-free survival) of KIF26B across all the tumors in TCGA [15]. The threshold values for distinguishing groups with low or high expression were defined as cutoff-high (50%) or cutoff-low (50%), respectively. Survival plots were acquired from GEPIA2. Of them, two curves were compared by the log-rank test.

### 2.3. Genetic Alterations

Data about KIF26B genetic alteration frequency and copy number and mutation type were retrieved through cBioPortal [16]. The global, disease-free, and progression-free survival information was obtained from the “Comparison” module. This included Kaplan–Meier schemes with log-rank *P* values of the TCGA cancer cases according to whether they had KIF26B genetic alterations.

### 2.4. Immune Cell Infiltration Analysis

We explored how the levels of KIF26B expression could be related to the infiltrating immune cells, such as endothelial and cancer-associated fibroblast cells. For this purpose, we utilized the “Immune-Gene” unit of TIMER2 and evaluated different cancers. To make immune infiltration estimations, the algorithms QUANTISEQ, XCELL, MCPCOUNTER, EPIC, TIMER, CIBERSORT, and CIBERSORT-ABS were applied. *P* values and partial correlation (cor) values were determined by Spearman's rank correlation test, purity-adjusted. The outcomes were displayed as a scatterplot and a heatmap.

### 2.5. KIF26B-Related Gene Enrichment Analysis

First, KIF26B-binding proteins were identified on the STRING website (https://string-db.org/) by searching for “KIF26B.” Then, the first 100 KIF26B-targeting genes were included in a Pearson correlation analysis that was based on the expression records of all TCGA tumor and normal tissues in GEPIA2. Moreover, a heatmap with the partial correlation (cor) index and *P* value of those genes was obtained using the “Gene_Corr” component of TIMER2. Lastly, the Venn diagram viewer allowed the observation of the KIF26B-binding and interacting genes [17].

## 3. Results

### 3.1. Gene Expression Analysis

**Figure 1:**
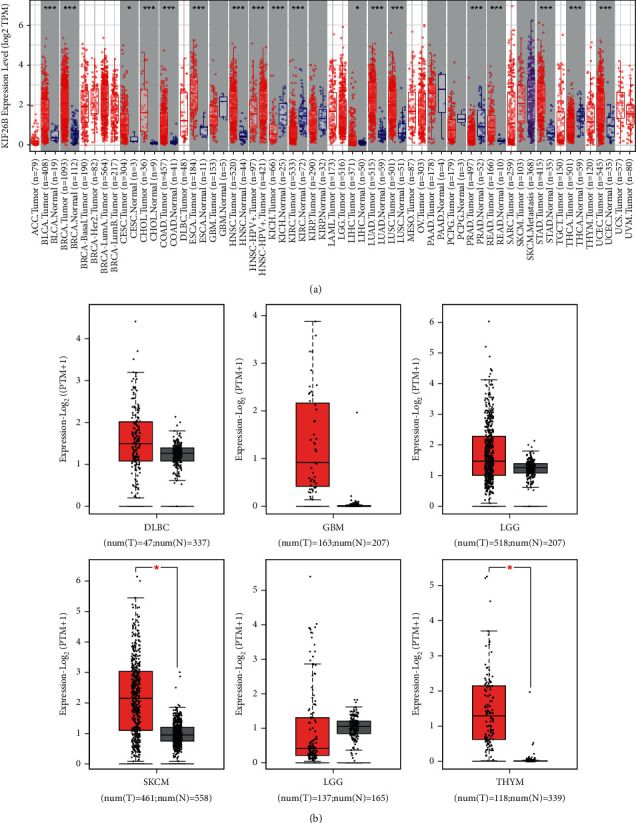
Expression levels of KIF26B in diverse cancers. (a) TIMER2 analysis of KIF26B expression in specific cancer types. (b) Box plot data of SKCM, THYM, DLBC, GBM, LGG, and TGCT in TCGA cohorts when compared to healthy tissues in the GTEx records. ^*∗*^*P* < 0.05.

The expression level of KIF26B in BLCA (bladder urothelial carcinoma), BRCA (breast invasive carcinoma), CHOL (cholangiocarcinoma), CESC (cervical squamous cell carcinoma and endocervical adenocarcinoma), GBM (glioblastoma multiforme), COAD (colon cancer), ESCA (esophageal carcinoma), HNSC (head and neck squamous cell carcinoma), HNSC-HPV+, KICH (kidney chromophobe), KIRC (kidney clear cell carcinoma), KIRP (kidney papillary cell carcinoma), LIHC (liver cancer), LUAD (lung adenocarcinoma), LUSC (lung squamous cell cancer), PRAD (prostate cancer), READ (rectum adenocarcinoma), STAD (stomach cancer), THCA (thyroid cancer), and UCEC (uterine corpus endometrial carcinoma) was higher than in the tumor-adjacent tissues (Figure 1(a)).

We further explored whether KIF26B was differentially expressed in tumor and normal tissues. The GTEx database was used as the controls. In SKCM (cutaneous melanoma) and THYM (thymoma), KIF26B was highly expressed in tumor tissues (*P* < 0.05). No significant differences were detected between DLBC (diffuse large B-cell lymphoma), GBM, LGG (brain glioma, lower grade), and TGCT (testicular germ tumor) and their corresponding normal samples (Figure 1(b), *P* < 0.05). The relationship between KIF26B expression and pathological stages of cancers was analyzed via the “Stage Plot” component of GEPIA, and strong positive correlations were found for BLCA, KICH, LIHC, and PAAD (*P* < 0.05) (Figure S1).

**Figure 2:**
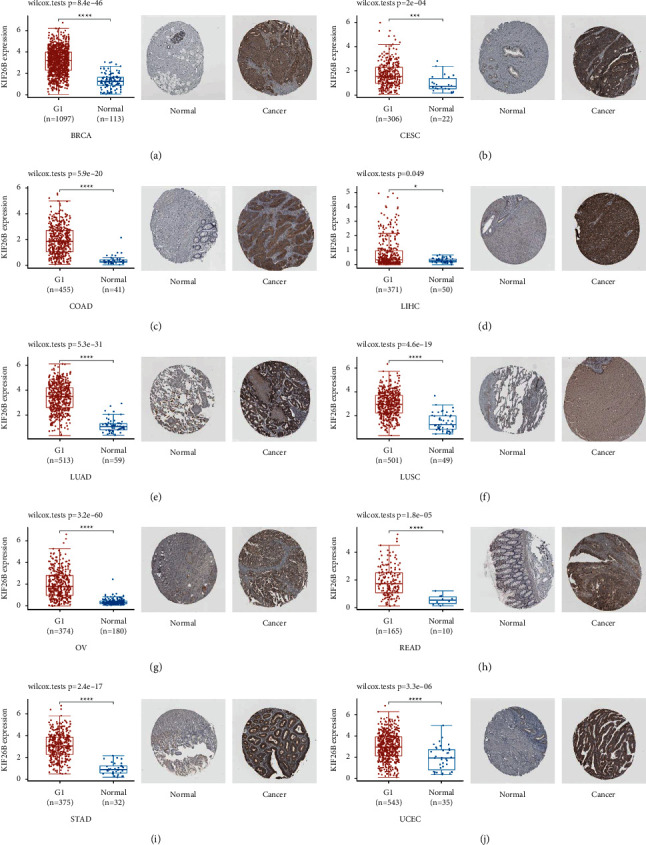
Comparison of KIF26B gene expression in tumor and healthy tissues within the TCGA dataset (left). Immunohistochemistry images of normal (middle) and tumor (right) tissues. (a) BRCA. (b) CESC. (c) COAD. (d) LIHC. (e) LUAD. (f) LUSC. (g) OV. (h) READ. (i) STAD. (j) UCEC.

Furthermore, the HPA database was consulted to analyze the IHC results of KIF26B. The gene expression data obtained from TCGA were used for comparisons. After the assessments, the results from the two databases were consistent with each other. In BRCD, CESC, COAD, LIHC, LUAD, LUSC, OV (ovarian serous cystadenocarcinoma), READ, STAD, and UCEC, the expression of KIF26B was higher than the equivalent normal tissues (*P* < 0.05) (Figure 2).

### 3.2. Survival Analysis

**Figure 3:**
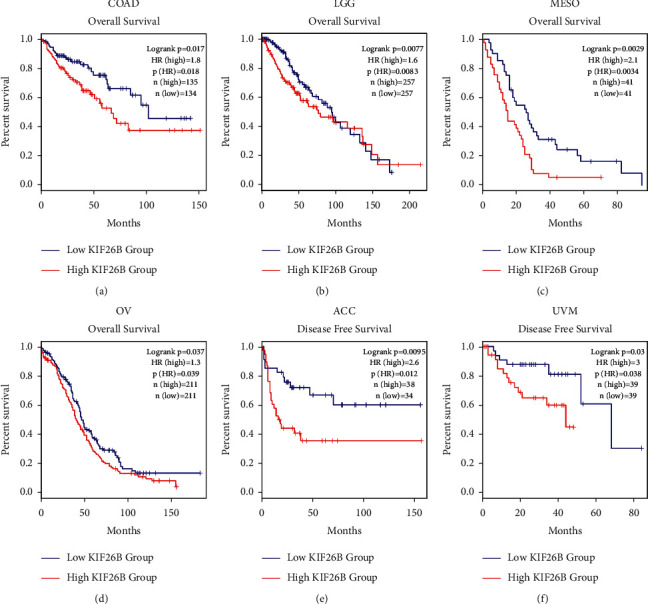
Kaplan–Meier analysis of KIF26B in different cancers. (a–d) Overall survival (OS) of COAD, LGG, MESO, and OV in groups with low or high KIF26B expression. (e, f) Disease-free survival (DFS) of ACC and UVM in groups with low or high KIF26B expression.

Correlations between prognosis and KIF26B gene expression in various cancers were studied using the GEPIA dataset. Overall survival (OS) and disease-free survival (DFS) values were assessed with the TCGA and GEO datasets. Cancer cases were categorized into low and high-KIF26B expression groups. As shown in Figures 3(a)–3(d), OS was poorer in the high-KIF26B group, with COAD (*P*=0.018), LGG (*P*=0.0083), MESO (*P*=0.0034), and OV (*P*=0.039). On the contrary, low KIF26B was linked to longer DFS in ACC (*P*=0.012) and UVM (*P*=0.03), compared to the high-KIF26B expression group (Figures 3(e) and 3(f)).

### 3.3. Genetic Alteration of KIF26B in Different Cancers

**Figure 4:**
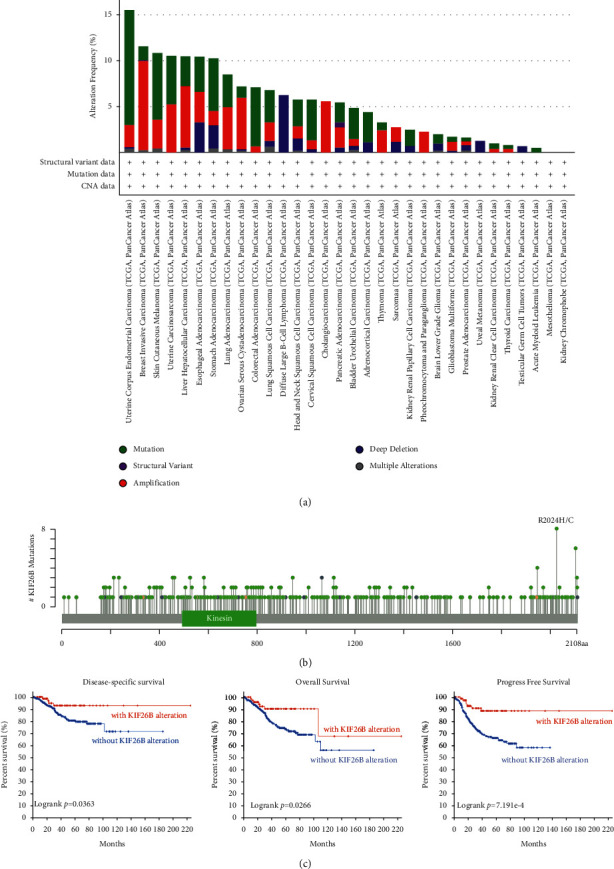
Gene alteration of KIF26B in TCGA cancers. cBioPortal was used to analyze the mutation features of cancer in TCGA. (a) Frequency of the mutation type. (b) Mutation site. (c) The potential relationship between genetic alterations of KIF26B and clinical prognosis of patients with various cancers.

Genomic mutation has been widely associated with cancerogenesis [18]. In this study, we analyzed genetic alterations of KIF26B in cancers within the TCGA cohorts. KIF26B was the more frequent mutation (>10%) in patients with esophageal adenocarcinoma, UCS (uterine carcinosarcoma), UCEC, BRCA, SKCM, LIHC, and STAD. On the contrary, the alteration frequencies were highest in esophageal adenocarcinoma, BRCA, UCS, LIHC, OV, and CHOL (>5%; Figure 4(a)). Special attention was drawn towards the CHOL cases (amplification ∼7%; Figure 3(a)). The types, sites, and case number of KIF26B genetic variations are shown in Figure 4(b). Furthermore, potential correlations between genetic alterations of KIF26B and clinical prognosis in different cancers were tested. As shown in Figure 4(c), when a KIF26B mutation was present in UCEC, there was a better prognosis for disease-specific survival (*P*=0.0363), overall survival (*P*=0.0266), and progression-free survival (*P* < 0.01).

### 3.4. KIF26B Protein Phosphorylation

**Figure 5:**
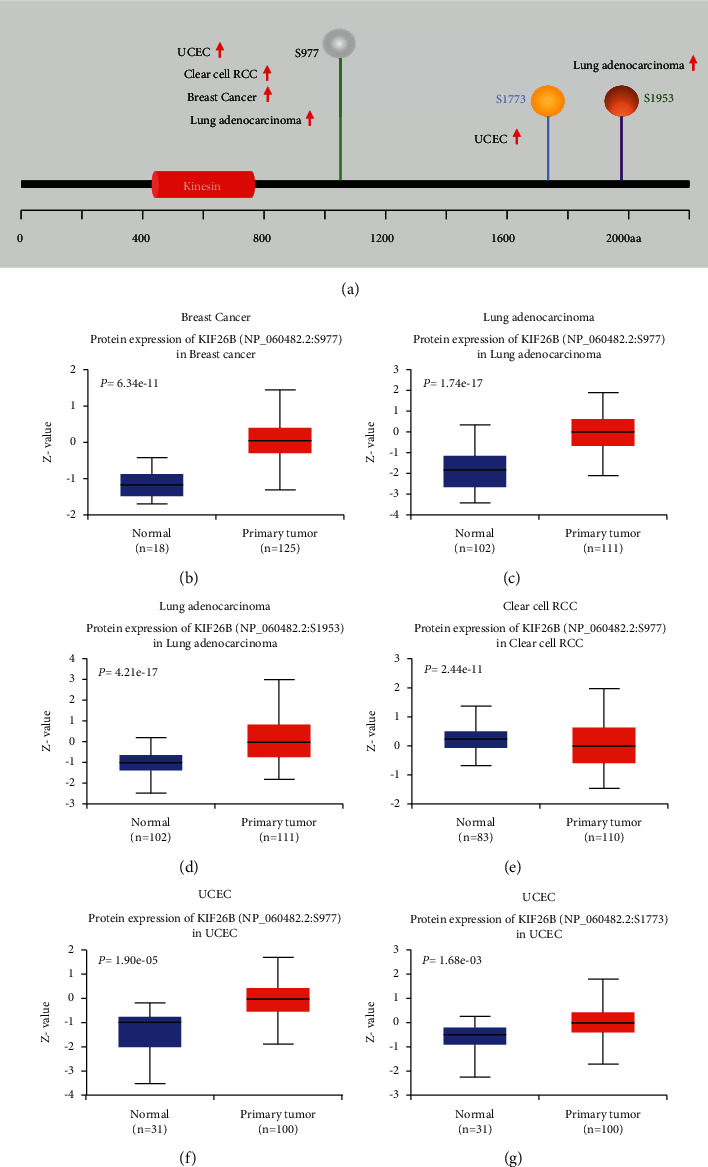
Protein phosphorylation in KIF26B. The differences in KIF26B protein phosphorylation between normal tissues and tumor samples were analyzed through the CPTAC dataset. A summary of the KIF26B phosphorylation sites and the significant differences found (a). Box plots of specific phosphorylation sites in a variety of cancers, including breast cancer (b), lung adenocarcinoma (c, d), clear cell RCC (e), and UCEC (f, g).

The phosphorylation levels of the KIF26B protein between tumor samples and normal tissues were compared. Four types of tumors were analyzed using the CPTAC dataset, including breast cancer, KIRC, LUAD, and UCEC. A summary of the phosphorylation sites of KIF26B and the detected differences between tumor and healthy tissues is shown in Figure 5(a). In all four types of tumors, the S977 site exhibited a higher phosphorylation level compared to normal tissues. Furthermore, in breast cancer, the KIF26B phosphorylation level in sites S1006, S1081, S1724, S1772, and S1979 was higher than in normal tissues, and so was site S1953 in LUAD and site S1773 in UCEC (Figures 5(b)–5(g)).

### 3.5. Immune Infiltration

**Figure 6:**
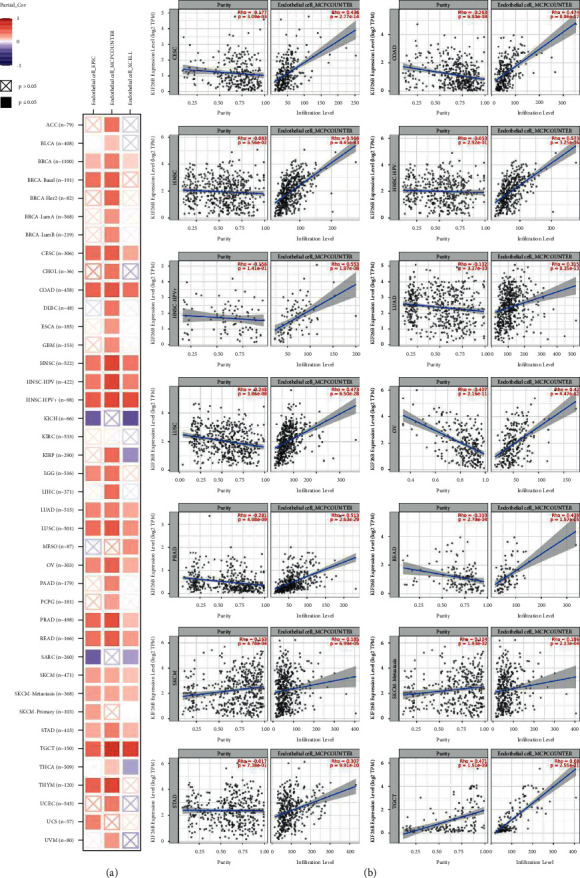
KIF26B expression and immune infiltration of endothelial cells: correlation analysis. The XCELL, MCPCOUNTER, and EPIC algorithms were used to estimate potential associations in various TCGA cancer types.

**Figure 7:**
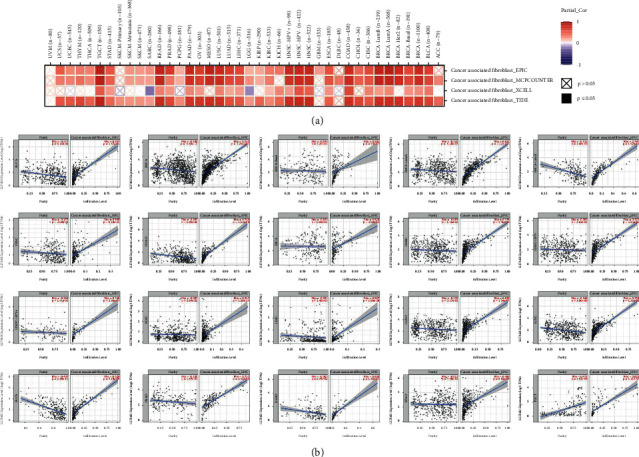
KIF26B expression and immune infiltration in cancer-associated fibroblasts: correlation analysis. The XCELL, MCPCOUNTER, and EPIC algorithms were used to estimate potential associations in various TCGA cancer types.

Immune cells that infiltrate tumors are one of the major components in the tumor microenvironment and play a crucial part in the initiation, development, and metastasis of cancer [19]. Lu et al. suggested that endothelial cells stimulate colorectal cancer stem cells in a paracrine or juxtacrine fashion [20]. Moreover, Du et al. reported that the cancer-associated fibroblast gene CALD1 may influence bladder cancer progression by modulating the tumor microenvironment [21]. Therefore, a possible association between the grade of immune infiltration and the gene expression of KIF26B in various cancer types was investigated. The analysis was performed through the TCGA database with the XCELL, MCPCOUNTER, EPIC, and TIDE algorithms. The results revealed a positive correlation between the expression of KIF26B and the immune infiltration of endothelial cells in CESC, COAD, HNSC (HPV (human papillomavirus) +/−), LUAD, LUSC, OV, PRAD, READ, SKCM, SKCM-metastasis, STAD, and TGCT (Figure 6). There were also positive correlations between the expression of KIF26B and the infiltration of cancer-associated fibroblasts (CAFs) in TCGA tumors, including ACC, BLCA, BRCA (BRCA-LumA, BRCA-LumB, and BRCA-basal), CESC, COAD, ESCA, HNSC (HPV (human papillomavirus) +/−), KIRC, LIHC, LUAD, LUSC, OV, PAAD, PRAD, READ, STAD, and TGCT (Figure 7(a)). To present the scatterplot data of the tumors, a single algorithm was used (Figure 7(b)). For instance, the positive association between KIF26B expression and the infiltration grade of CAFs (Figure 7(b)), cor = −0.627, *P* = 1.57*e* − 41) was discovered via the MCPCOUNTER algorithm in BLCA.

### 3.6. GO and KEGG Enrichment Analyses of KIF26B-Related Genes

**Figure 8:**
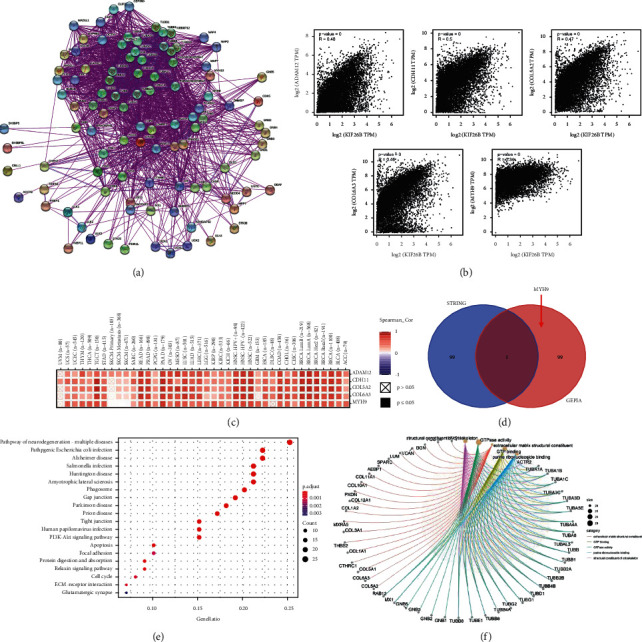
Gene enrichment analysis of KIF26B. The STRING database was used to identify KIF26B-binding proteins (a). The expression of KIF26B-correlated genes was explored by TCGA and/or GTEx via the GEPIA2 tool. The KIF26B expression level was positively correlated to that of Adam12, CDH11, COL5A2, COL6A3, and MYH9 (b). Heatmap of five KIF26B-correlated genes from a variety of cancer types (c). The intersection of KIF26B-binding and interacting genes after selection by Venn diagram analysis (d). KEGG and GO enrichment analyses were performed following the KIF26B-binding and interacting genes' data (e, f).

To further inquire into the molecular mechanism of KIF26B in tumorigenesis and development, pathway enrichment analysis of KIF26B-correlated genes and KIF26B-binding proteins was performed. Protein-protein interaction (PPI) networks were constructed based on a total of 100 KIF26B-binding proteins found in the Proteins (STRING) database (Figure 8(a)). Additionally, KIF26B-correlated genes were explored by TCGA and/or GTEx expression data via the GEPIA2 tool. The results indicated that the expression of KIF26B was positively correlated to that of genes Adam12 (Adam metallopeptidase domain 12; *R* = 0.48), CDH11 (cadherin-11; *R* = 0.5), COL5A2 (collagen type V alpha 2 chain; *R* = 0.47), COL6A3 (collagen type VI alpha 3 chain; *R* = 0.46), and MYH9 (myosin heavy chain 9; *R* = 0.34) (all *P* < 0.001; Figure 8(b)). As displayed in Figure 8(c), the expression of KIF26B also had a positive correlation with the above five genes in a variety of cancer types. Only one common gene (MYH9) in the intersection of the above two groups was selected by means of a Venn diagram (Figure 8(d)).

KEGG and GO enrichment analyses were conducted on the two datasets. The KEGG results indicated that KIF26B-related genes may affect tumor pathogenesis by “PI3K-AKT signaling,” “apoptosis,” and “cell cycle” pathways (Figure 8(e)). The GO enrichment data (Figure 8(f)) showed that most of these genes were connected with “extracellular matrix structural constituent,” “GTP binding,” “GTPase activity,” “purine ribonucleoside binding,” and “structural constituent of the cytoskeleton.”

## 4. Discussion

Previous studies have suggested that high expression of KIF26B is an indicator of poor prognosis in various cancers. KIF26B is relevant to cell proliferation, migration, invasion, and even drug resistance in cancer [22]. Zhang et al. [3] reported that KIF26B may contribute to multiple malignancies via the VEGF pathway. Nevertheless, specific genes may be expressed and function differently in disparate tumors due to tumor heterogeneity [23]. Whether KIF26B could play a role in the growth of diverse tumors through particular molecular mechanisms remains to be explored. As of now, no pan-cancer studies from the perspective of various tumors have been available. In this work, we summarized the molecular features of the KIF26B gene in 33 different tumors through the TCGA, GEO, and CPTAC databases and predicted the functional mechanism of tumor occurrence and development.

Our analysis revealed that the gene expression of KIF26B was significantly higher in 23 types of tumor tissues than in adjacent normal tissues. The GTEx database was used as compensation for the higher expression of KIF26B in SKCM and THYM than in normal tissues. Furthermore, the IHC analysis confirmed this tendency at the protein level. The finding that the KIF26B gene has a universal high expression across various tumor tissues suggests the importance of a pan-cancer analysis. Recent studies have reported that KIF26B is overexpressed in BRCA [2], ECA [24], colorectal cancer [4], and gastric cancer [3]. The overexpression of the KIF26B gene is also closely related to poor prognosis among the above cancers, which matches the survival data results of the present work.

Nowadays, targeted therapy has been one of the most precise treatments for malignancies. It is widely known that numerous types of cancers are triggered by genetic alterations of one or more genes [25], and the prognosis of particular cancer may be determined by the way the gene has been altered [26]. These modifications include gene mutation, amplification, deep deletion, structural variants, and multiple alterations. Our analysis indicated that KIF26B mostly underwent mutation and amplification alterations, and KIF26B mutation in UCEC displayed longer DFS, OS, and PFS. The development of the gene-editing technology in the near future would allow the modification of the KIF26B gene, which could be a promising method for curing cancers such as UCEC, BRCA, SKCM, UCS, LIHC, and STAD. On the contrary, we found the KIF26B protein phosphorylation level was higher in tumor tissues compared to normal tissues, while different tumors shared diverse phosphorylation sites. These findings imply the great importance of clarifying the phosphorylation sites of a certain cancer type and that precise targeting of KIF26B during treatment would have a personal character depending on the genetic diagnosis.

Given the emergent concept of tumor immune microenvironment (TME), significant pan-cancer analysis was conducted. This study evidenced that the expression of KIF26B and the immune infiltration of endothelial cells were positively correlated in most cancers, except for COAD, SKCM, and SKCM-metastasis. Currently, immunotherapy has become a new antitumor strategy by monitoring and clearing tumor cells. In particular, endothelial cells are the most relevant cells in antitumor immunity and constitute an important fraction of the tumor infiltration lymphocytes [27]. Hence, the role and activity of endothelial cells are vital for immunotherapy responsiveness. In COAD, SKCM, and SKCM-metastasis, KIF26 was overexpressed, while the infiltration of the endothelial cells was expressed in the opposite tendency. Thus, targeting endothelial cells' activation or blocking the clearance escape of tumor cells could potentially improve the prognosis of patients with these cancers.

On the contrary, KEGG and GO analyses were carried out to explore the molecular mechanisms of KIF26B. The results showed that KIF26B-related genes, including Adam12, CDH11, COL5A2, COL6A3, and MYH9, were mainly enriched in “PI3K-AKT signaling,” “apoptosis,” and “cell cycle” pathways; this corresponds with previous studies [5, 28]. Our enrichment analyses indicated that KFI26B may exert its tumorigenic effects by promoting tumor growth, inhibiting cell apoptosis, and reordering the cell cycle.

In conclusion, this pan-cancer analysis found that KIF26B can act as an oncogene and can have genetic alterations associated with the diagnosis and prognosis of various cancers. Moreover, KIF26B can affect endothelial cells' infiltrating and molecular pathways in most cancers. This study may contribute to better understanding the role of KIF26B in cancerogenesis and development and provide a new horizon for more precise and personalized treatments for cancers.

## Data Availability

Publicly available datasets were used in this study. The data can be found in the gene in the TCGA (https://portal.gdc.cancer.gov), TIMER2 (http://timer.cistrome.org/), GEPIA2 (http://gepia2.cancer-pku.cn/#index), GTEx (https://www.gtexportal.org/), CPTAC (https://proteomics.cancer.gov/programs/cptac), and HPA (https://www.proteinatlas.org/).

## Supplementary Materials

Supplementary Materials Figure S1: relationship between KIF26B expression and pathological stages of cancers. For this analysis, the “Stage Plot” module of GEPIA was used considering pathological stages of BLCA (a), KICH (b), LIHC (c), and PAAD (d).
