# Supplementary material for: KIF26B in the Prognosis and Immune Biomarking of Various Cancers: A Pan-Cancer Study
Source: J Oncol. 2022 Mar 22;2022:4829697. doi: 10.1155/2022/4829697 (PMC8964195; doi:10.1155/2022/4829697)

**Figure S1**

Relationship between KIF26B expression and pathological stages of cancers. For this analysis, the “Stage Plot” module of GEPIA was used considering pathological stages of BLCA (a), KICH (b), LIHC (c), and PAAD (d).


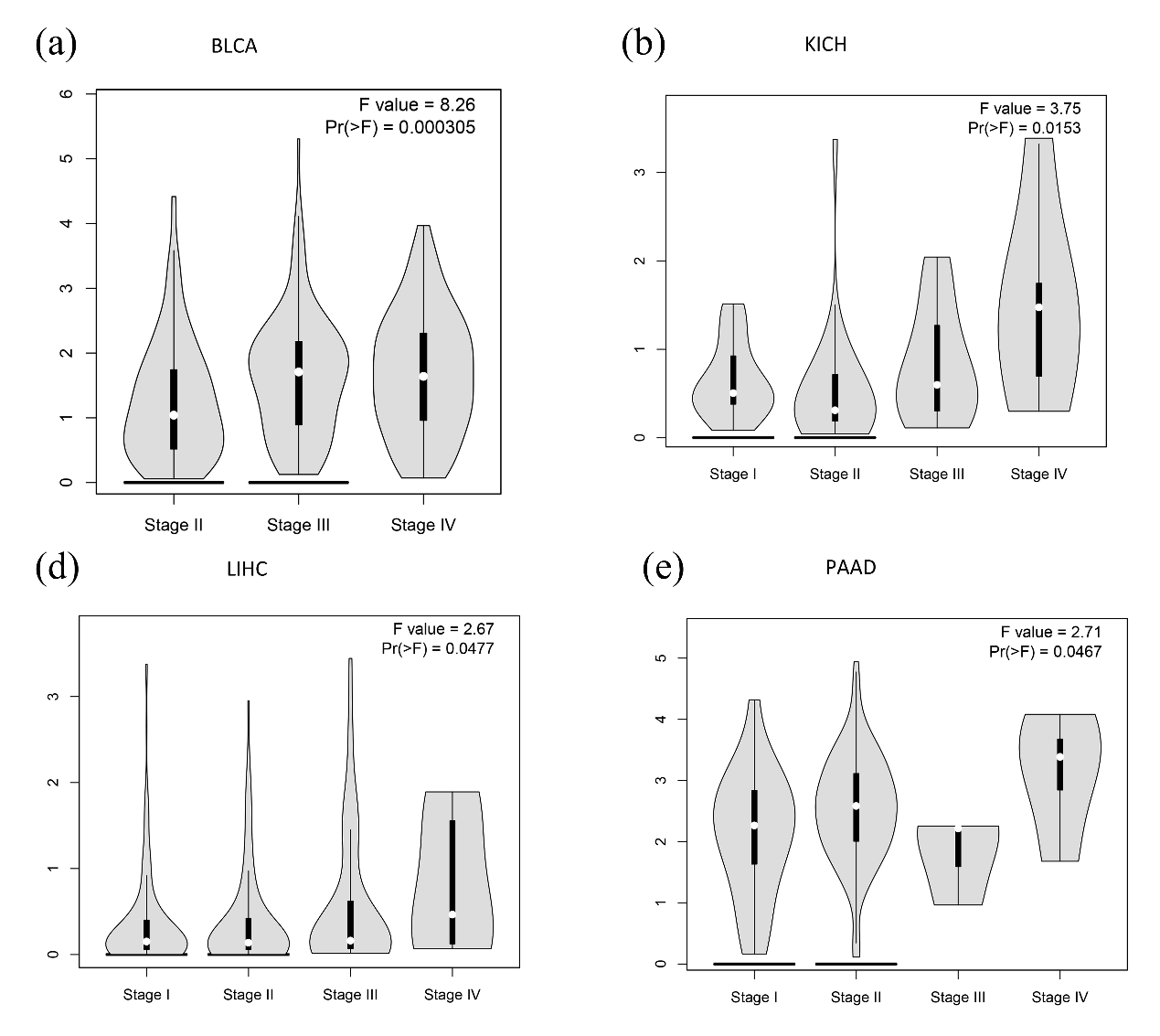

Supplement: Supplementary Materials — Figure S1: relationship between KIF26B expression and pathological stages of cancers. For this analysis, the “Stage Plot” module of GEPIA was used considering pathological stages of BLCA (a), KICH (b), LIHC (c), and PAAD (d). [file 4829697.f1.docx]
